# High prevalence of s*pa* type t571 among methicillin-susceptible *Staphylococcus aureus *from bacteremic patients in a French University Hospital

**DOI:** 10.1371/journal.pone.0204977

**Published:** 2018-10-09

**Authors:** Isabelle Bonnet, Brune Millon, Hélène Meugnier, François Vandenesch, Max Maurin, Patricia Pavese, Sandrine Boisset

**Affiliations:** 1 Laboratoire de Bactériologie, Grenoble-Alpes University Hospital, Grenoble, France; 2 Service de Maladies infectieuses, Grenoble-Alpes University Hospital, Grenoble, France; 3 Hospices Civils de Lyon, Laboratoire de Bactériologie, Centre de Biologie et de Pathologie Est, Bron, France; 4 CIRI, Centre International de Recherche en Infectiologie, Université de Lyon, Lyon, France; 5 Inserm, U1111, Lyon, France; 6 Ecole Normale Supérieure de Lyon, Lyon, France; 7 Université Lyon 1, Lyon, France; 8 CNRS, UMR 5308, Lyon, France; 9 TIMC-TheREx Laboratory UMR 5525, CNRS, Grenoble-Alpes University Hospital, Grenoble, France; Rockefeller University, UNITED STATES

## Abstract

*Staphylococcus aureus* bacteremia is one of the most frequent severe bacterial infections worldwide, with an associated mortality of about 20–40% in developed countries. In 2013, we noted an increase in this infection in the teaching hospital in Grenoble, France, compared to 2012. The mean incidence of *S*. *aureus* bacteremia was 0.28 per 1,000 patient-days in 2012 and 0.35 per 1,000 patient-days in 2013. This trend was confirmed in 2014 (0.35 per 1,000 patient-days). In the present work we aimed to study the population of patients presenting with *S*. *aureus* bacteremia in 2013 and to genotype the corresponding *S*. *aureus* strains in order to identify a successful and/or virulent genotype to design a specific infection control program. One hundred ninety-one *S*. *aureus* isolates (including 9 methicillin-resistant) out of 199 corresponding cases of bacteremia were characterized with the *spa* typing method. Among 108 *spa* types, t571, t002, t008 and t084 were the most prevalent. Although not widely prevalent, t571 was the most frequently identified clone (8.4% of all isolates). *Spa* type t571 has been described in previous studies as belonging to the clonal complex CC398, which is consistent with the recent emergence of methicillin-susceptible *S*. *aureus* CC398 reported in blood cultures in Europe.

## Introduction

*Staphylococcus aureus* represents the second leading cause of community- and healthcare-associated bacteremia in developed countries with an overall annual incidence rate of 15–40 per 100 000 population [1–4]. A 30-day mortality of 20% has been reported in developed countries [5]; therefore, the identification of the factors influencing its rise, both genetic and/or environmental, is of major importance for the prevention of this infection. The distribution of methicillin-resistant *S*. *aureus* (MRSA) clonal types has been well characterized across different geographic regions and care settings. Although methicillin-susceptible *S*. *aureus* (MSSA) accounts for a large proportion of staphylococcal infections, comparatively little is known about its molecular epidemiology, in particular in France. *S*. *aureus* bacteremia, regardless of methicillin-susceptibility, is a worldwide problem associated with increased morbidity and mortality among hospitalized patients. An epidemiological study conducted in a cohort of French hospitals allowed the identification of first cases of blood infections due to *S*. *aureus* ST398 [6]. Initially, the ST398 was a MRSA clone associated with livestock carriage in pig farms. Subsequently, these clones spread to human with a worldwide dissemination [7, 8]. Several human infections of MRSA CC398, including bacteremia, have been documented, sometimes without livestock contact [9, 10]. Methicillin-susceptible variants of CC398 have been occasionally described and phylogenetic studies suggest that livestock-associated MRSA CC398 emerged from human MSSA CC398 [11]. Several studies have shown that MSSA CC398 isolated in humans was not livestock associated [6, 12]. Many *spa* types have been associated with the CC398 background [13], but the assignation of *spa* t571 to CC398 has been repeatedly observed [13, 14, 15].

In the Grenoble-Alpes University Hospital, France, the mean incidence of *S*. *aureus* bacteremia cases rose from 0.28 per 1,000 patient-days in 2012 to 0.35 per 1,000 patient-days in 2013. This trend was confirmed in 2014 (0.35 per 1,000 patient-days). To investigate this alarming observation, clinical data on all cases of *S*. *aureus* bacteremia in 2013 were collected and corresponding isolates were characterized by *spa* typing, as this molecular typing method is accurate, simple, cost-effective, rapid and easily interpretable [16, 17].

## Material and methods

### Study population

All consecutive patients hospitalized in Grenoble-Alpes University Hospital (2000 beds) with *S*. *aureus* bacteremia diagnosed between 1^st^ January 2013 and 31^st^ December 2013 were retrospectively enrolled. *S*. *aureus* bacteremia was defined as patients with at least one blood culture bottle, performed with the Bactec system (Becton Dickinson, USA) that was positive for *S*. *aureus*. Study ethics approval was obtained from Center for Ethics Committee (CECIC) in Rhône-Alpes-Auvergne District (agreement n°IRB 5891).

Clinical and therapeutic data were retrospectively collected from the patient’s medical records. Clinical data included demographics, background characteristics (comorbidities, immunocompromised status), and healthcare contacts before hospitalisation. Characteristics of *S*. *aureus* bacteremia comprised duration, and associated infection site (endocardium, lung, bones and joints, muscles, urine). Therapeutic data consisted of antibiotics, catheter removal, surgery, and admission to an intensive care unit. A healthcare-associated infection could be either nosocomial or non-nosocomial. An infection was considered nosocomial if *S*. *aureus* bacteremia developed in a patient hospitalized at least 48 hours prior to the onset of signs/symptoms indicating bacteremia. A non-nosocomial healthcare–associated infection was defined as *S*. *aureus* bacteremia diagnosed within 48 hours of admission in a patient with extensive healthcare contact as reflected by any of the following criteria: (a) received intravenous therapy, wound care, or specialized nursing care at home within the 30 days prior to the onset of *S*. *aureus* bacteremia; (b) attended a hospital or hemodialysis clinic or received intravenous chemotherapy within the 30 days before the onset of *S*. *aureus* bacteremia; (c) was hospitalized in an acute care hospital for 2 or more days in the 90 days before the beginning of *S*. *aureus* bacteremia; or (d) lived in a nursing home or long-term care facility. Community-acquired infection was identified as *S*. *aureus* bacteremia within 48 hours of admission with no criteria for healthcare-associated infection.

The diagnosis of infective endocarditis (IE) was classified as definite, possible or was excluded according to modified Duke’s criteria [18].

### *S*. *aureus* isolates

Bacteremia-associated *S*. *aureus* strains were routinely identified on the basis of their colony morphology, Gram staining, catalase test and slide test for clumping factor (Staphytect Plus; Oxoid, UK). All identifications were confirmed by MALDI-TOF MS, using a Microflex LT instrument, Flexcontrol 3.0 software and the Biotyper 2.0 database (Bruker Daltonics, USA). Following antimicrobial susceptibility testing (see below), all isolates were stored at room temperature in agar tubes (BioRad, USA).

### Antimicrobial susceptibility-testing

Susceptibility-testing was performed on Mueller-Hinton agar plates by the disk diffusion method (antibiotic disks; Bio-Rad, USA), according to French guidelines for antibiogram (CA-SFM, 2015). Antibiotics tested were cefoxitin, kanamycin, tobramycin, gentamicin, netilmycin, amikacin, doxycycline, erythromycin, lincomycin, pristinamycin, linezolide, rifampicin, cotrimoxazole, ofloxacin, fusidic acid, fosfomycin, vancomycin, and teicoplanin. The detection of methicillin resistance has been determined with the cefoxitin disk screen test in accordance with EUCAST recommendations.

### *Spa* sequencing

*Spa* typing was performed with the Ridom Staph Type standard protocol [19], by using an in house procedure with an ABI 3130xl sequencer (Applied, USA) for double stranded DNA sequencing and by using Ridom Staph Type software (version 1.5) [20] (http://spaserver.ridom.de/), which automatically analyses *spa* repeats and assigns *spa* types. *Spa* types were analysed using the integrated based upon repeat pattern (BURP) algorithm [21].

### Characterization of spa t571 strains

For all *spa* t571 strains, a PCR assay was performed to detect the CC398-specific *sau1-hsdS1* variant [22]. Erythromycin-resistant isolates were screened with primers specific for the *ermT* gene as previously described [23].

## Results

### Strain characteristics

Over the study period, 199 patients with *S*. *aureus* bacteremia were included. Among the 199 corresponding strains, two were not stored, six did not recover after storage, leaving 191 strains for further characterization. Of the 191 strains that we typed, the overall MRSA rate was 4.7% (*n* = 9) and the major resistances of the 191 isolates were to erythromycin (*n* = 39, 20.4%) and ofloxacin (*n* = 12, 6.3%) (Table 1). All the isolates were susceptible to vancomycin. Among the 191 characterized isolates, MRSA were detected in 5.3% (*n* = 8) of the healthcare-associated (HCA) infections (*n* = 151) and in 2.5% (*n* = 1) of the community-acquired (CA) infections (*n* = 40). The resistance of MRSA towards fluoroquinolones was 88.9%.

**Table 1:** Antibiotic resistance profiles of 191 isolates of *S*. *aureus* collected in 2013 in Grenoble-Alpes University Hospital, France.

	MSSA (n = 182)		MRSA (n = 9)		t571 (n = 16)	
	S	R (%)	S	R (%)	S	R (%)
Antibiotic						
Oxacillin	182	0	0	9 (100)	16	0
Gentamicin	181	1 (0.5)	9	0	16	0
Tobramycin	181	1 (0.5)	5	4 (44.4)	16	0
Kanamycin	179	3 (1.6)	4	5 (55.6)	16	0
Amikacin	180	2 (1.1)	6	3 (33.3)	16	0
Netilmycin	181	1 (0.5)	9	0	16	0
Doxycycline	178	4 (2.2)	9	0	16	0
Erythromycin	145	37 (20.3)	6	3 (33.3)	4	12 (75)
Lincomycin	178	4 (2.2)	7	2 (22.2)	16	0
Cotrimoxazole	181	1 (0.5)	9	0	16	0
Fosfomycin	182	0	9	0	16	0
Fusidic acid	182	0	9	0	16	0
Pristinamycin	182	0	9	0	16	0
Rifampicin	182	0	8	1 (11.1)	16	0
Nitrofurantoin	182	0	9	0	16	0
Ofloxacin	178	4 (2.2)	1	8 (88.9)	16	0
Linezolid	182	0	9	0	16	0
Teicoplanin	182	0	9	0	16	0
Vancomycin	182	0	9	0	16	0

**Fig 1:**
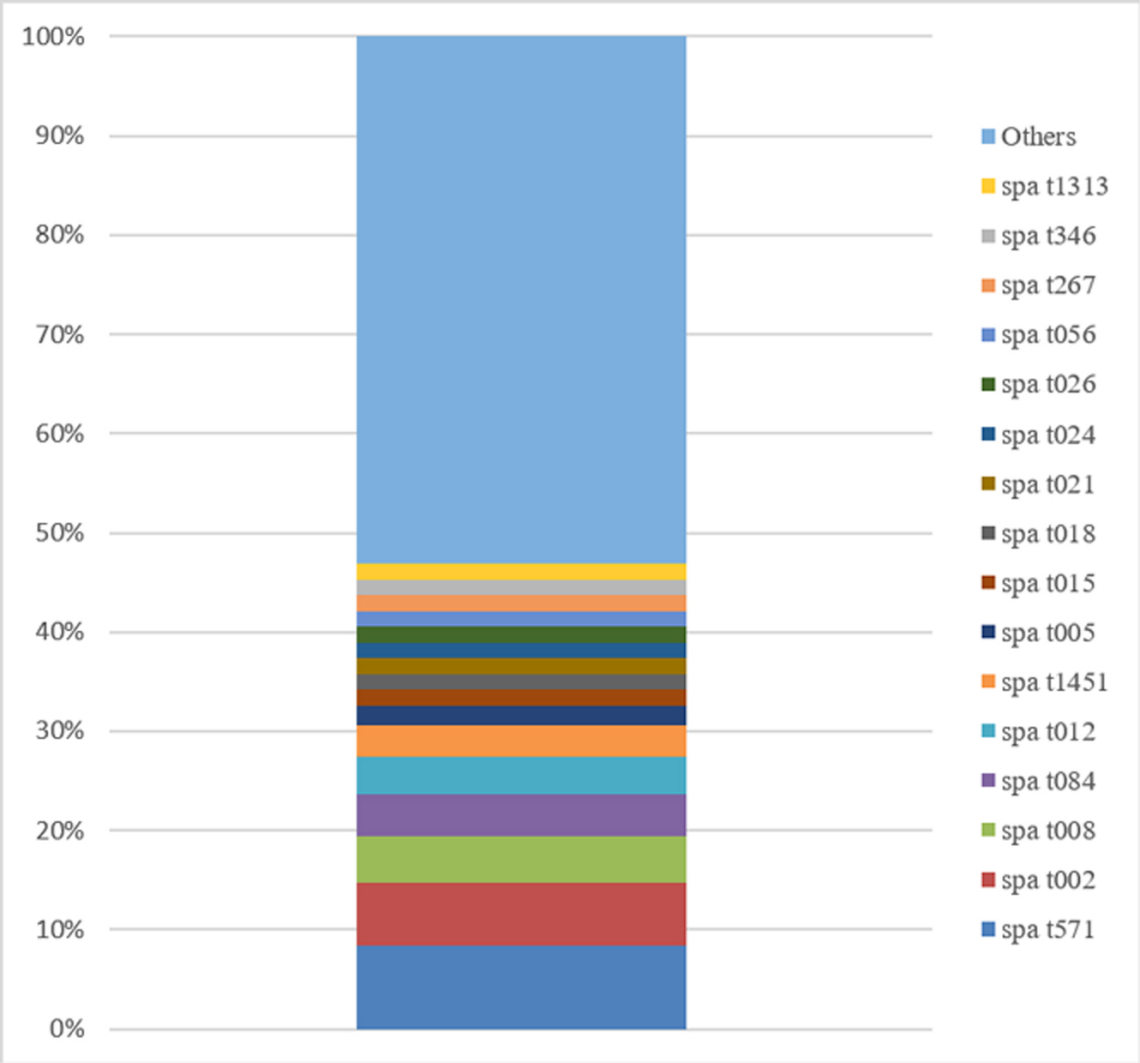
Overall frequency of *spa* types. Types found in ≤2 isolates are categorized as “others” (*n* = 92 of 108 total *spa* types).

Among the 191 *S*. *aureus* isolates examined, 108 *spa* types were identified, two of which were newly assigned during this study. One of the 191 isolates could not be assigned to a *spa* type through the protocol described above and was consequently excluded from clustering analyses. For two patients, two *S*. *aureus* isolates were identified corresponding to two different *spa* types. The frequency of each *spa* type was variable, with 1 to 16 isolates per *spa* group type. Eighty-one *spa* types were represented by a single isolate, while 27 were represented by at least two isolates. The most prevalent *spa* type was t571, which accounted for 16 isolates (8.4% of all isolates). The 16 isolates were assigned to CC398 using the PCR targeting the CC398-specific variant of *sau1-hsdS1*. Twelve of these isolates were resistant to erythromycin (75%) (Table 1). We showed that *ermT* gene was the most common mechanism responsible for erythromycin resistance in these isolates, with 11 of the 12 strains positive for the *ermT* specific PCR (92%). The other most prevalent *spa* types were t002 (*n* = 12, 6.3%), t008 (*n* = 9, 4.7%) and t084 (*n* = 8, 4.2%). Among MRSA isolates, t008 was the most frequently detected (*n* = 5). The other *spa* types detected in MRSA isolates were t024 (*n* = 2), t080 (attributed most likely to the European-ST80 CA MRSA lineage) (*n* = 1) and t15255 (*n* = 1). Fig 1 shows the prevalence of the *spa* types within our collection. Using the integrated BURP algorithm, the 108 *spa* types were divided into 15 *spa* clusters and 19 singleton *spa* types. The prevalence of each *spa* cluster is noted in Table 2. Eleven strains were excluded because the number of repeats was less than five. The four major clusters were *spa* CC t015, CC t012, CC t024 and CC t002 and accounted for 42.6% (*n* = 46) of the *spa* types and 53.4% (*n* = 102) of the strains. *Spa* CC t015 was the most frequently detected for strains associated with infective endocarditis. *Spa* CC t015 was most prevalent for strains isolated in bone and joint infections, in skin and soft tissue infections and in infections of intravascular devices. *Spa* CC t012 was mostly detected for strains associated with respiratory infections and urinary tract infections.

**Table 2:** Frequencies of *spa* clusters. CA: Community-acquired infections; HCA: Healthcare-associated infections; IE: Infective endocarditis; severe infections: intensive care unit admission, septic shock and death.

Cluster (most common *spa* types)	No. of *spa* types (%) (n = 108)	No. of strains (%) (n = 191)	No. of CA strains (%) (n = 40)	No. of HCA strains (%) (n = 151)	No. of IE strains (%) (n = 8)	No. of strains associated with severe infections (%) (n = 64)
t015 (t015, t050, t230, t571, t1451, t4896)	22 (20.4)	46 (24.1)	12 (30)	35 (23.2)	3 (37.5)	15 (23.4)
t012 (t012, t018, t021)	10 (9.3)	20 (10.5)	4 (10)	16 (10.6)	0	11 (17.2)
t024 (t008, t024, t1171)	7 (6.5)	18 (9.4)	1 (2.5)	17 (11.3)	1 (12.5)	3 (4.7)
t002 (t002)	7 (6.5)	18 (9.4)	4 (10)	14 (9.3)	1 (12.5)	5 (7.8)
t189/3380 (t267)	6 (5.6)	8 (4.2)	1 (2.5)	7 (4.6)	0	3 (4.7)
t216 (t216)	5 (4.6)	6 (3.1)	0	4 (2.6)	0	2 (3.1)
t084 (t084, t279, t346)	5 (4.6)	15 (7.9)	5 (12.5)	10 (6.6)	0	5 (7.8)
t005 (t005)	4 (3.7)	7 (3.7)	2 (5)	5 (3.3)	1 (12.5)	3 (4.7)
t167	3 (2.8)	3 (1.6)	0	2 (1.3)	0	2 (3.1)
t127 (t177)	3 (2.8)	4 (2.1)	1 (2.5)	3 (2)	0	2 (3.1)
Other *spa* types	36 (33.3)	46 (24.1)	10 (25)	38 (25.2)	2 (25)	13 (20.3)

### Patient characteristics

As mentioned above, 199 patients with *S*. *aureus* bacteremia were included. The main characteristics of patients are shown in Table 3.

**Table 3:** Main characteristics of the 199 patients with *S*. *aureus* bacteremia and of the 16 patients infected with *spa* t571 strains.

		n (%)	t571 (n = 16)
Median age (years)		48 (range 0–95)	44 (range 0–87)
Male gender		131 (65.8)	10 (62.5)
Care unit	Medical	145 (72.9)	12 (75)
	Surgical	28 (14.1)	2 (12.5)
	Intensive care	26 (13.1)	2 (12.5)
Renal failure		49 (24.6)	3 (18.8)
Renal failure with hemodialysis		14 (7.0)	1 (6.3)
Diabetes mellitus		50 (25.1)	5 (31.3)
Immunosuppression		56 (28.1)	5 (31.3)
Associated infection site	Vascular foreign body	63 (31.7)	9 (56.3)
	Skin and soft tissue	36 (18.1)	1 (6.3)
	Bone and joint	32 (16.1)	3 (18.8)
	Lung	19 (9.5)	1 (6.3)
	Urinary tract	5 (2.5)	1 (6.3)
Infective endocarditis		8 (4)	0
Severe infections^a^		68 (34.2)	4 (25)
Presumed setting of acquired infection	Community	41 (20.6)	2 (12.5)
	Healthcare-associated	158 (79.4)	14 (87.5)
	Healthcare-associated nosocomial	105 (52.8)	6 (37.5)
	Healthcare-associated non nosocomial	53 (26.6)	8 (50)

^a^Intensive care unit admission, septic shock and death.

Most patients had severe comorbidities. Renal failure was reported in 24.6% of patients (*n* = 49). Immunosuppression was present in 28.1% of patients (*n* = 56). Only 4 patients (2%) were past or current injecting drug users. A deep focus of infection was detected in 86.9% of patients (*n* = 173). The most frequent deep focus of infection was an invasive vascular device, followed by skin and soft tissue and bone and joint localizations. Echocardiography (transthoracic or transoesophageal) was performed in 67.3% of patients and definite IE was diagnosed in 4% of patients (*n* = 8). Severe infections involving septic shock, admission to the intensive care unit and/or death concerned 68 patients (34.2%). The mortality rate was 21.6%.

*Spa* t571 bacteremia were associated with an infected vascular foreign body with 56.3% of the cases, which explain why 87.5% of these bacteremia are healthcare-associated infections.

## Discussion

In 2013, a large increase in *S*. *aureus*, mainly methicillin-susceptible, bacteremia was observed in our University Hospital compared to previous years (2012). Here we aimed to characterize all *S*. *aureus* isolates associated with bacteremia in 2013 in order to determine whether this increased prevalence could be attributed to the diffusion of an epidemic strain. Clinical data were also collected so as to determine any unknown risk factors for *S*. *aureus* bacteremia and interpret *spa* types according to clinical status.

The incidence of healthcare-associated *S*. *aureus* bacteremia was high (79.4%), as observed by Le Moing *et al*. [24]. Therefore, it seems essential to improve the prevention of these infections. In Australia the implementation of non-specific infection prevention and control initiatives coincided with a significant reduction in the incidence of healthcare-associated *S*. *aureus* bacteremia [25]. Several studies have shown that universal decolonization may also be appropriate, especially in the intensive care units [26, 27].

Only 8 cases of IE were noted in our population (4%). As echocardiography (transthoracic or transoesophageal) was not performed in 32.7% of patients, this estimation is probably conservative. In studies with more systematic use of echocardiography, the proportion of IE was higher with 15.6%, 22% and 19% reported respectively by Le Moing *et al*., Rasmussen *et al*. and Vos *et al*. [24, 28, 29]. This observation suggests the importance of actively searching for IE by performing transthoracic or transoesophageal echocardiography in cases of *S*. *aureus* bacteremia, as it is recommended in the 2009 European guidelines on IE. As no change in the population profile was clearly manifest compared to similar cohort studies conducted before 2013 [30–32], it seemed useful to study the genotype of *S*. *aureus* strains by *spa* typing. In our study, *spa* t571 accounts for 8.4% of our isolates and is the predominant *spa* type. The assignation of *spa* t571 to CC398 has been repeatedly observed [13, 14, 15, 33]. In our study, 100% of *spa* t571 strains belong to the CC398. Recently, Valentin-Domelier *et al*. highlighted the emergence of MSSA ST398 in bacteremia in the “Centre” region of France (2.5 million inhabitants), as has been reported worldwide [6, 34]. The first cases of ST398 bacteremia in French patients living in animal free environments were reported in 2007. The ST398 clone accounted for 15% of MSSA isolates responsible for bacteremia in 2015 whereas it had never been detected in bacteremia cases before 2007 [35]. MSSA CC398 isolates, harboring distinct characteristics from the livestock-associated multiresistant highly pathogenic clone, seem to be associated with high mortality in bacteremia and easy transmissibility [14, 36]. We note that ST398-*spa* t571 strains described so far exhibited the macrolide-lincosamide-streptogramin B resistance gene *ermT* [13]. In our study, 75% of the *spa* t571 strains were erythromycin-resistant and we demonstrated that the *ermT* gene conferred this resistance for 92% of the strains. This marker of the human clade [15] suggests that all CC398 MSSA likely correspond to the ancestral human population. Two other evidences argue in favor of this conclusion: theses strains are all MSSA and they are not resistant to tetracycline (a marker of LA-MRSA). The predominance of the clone *spa* t571 implies that our University Hospital is probably concerned by the spread of this clone. This is not the case for all European countries, e.g. in Germany where MSSA *spa* t571 constituted only 0.14% of German isolates from infections in human between 2006 and 2012 [15].

In contrast to the clustering usually noted with MRSA strains, the majority of our isolates, which were methicillin susceptible, showed broad genetic diversity. Similar MSSA heterogeneity has been reported across Europe and in the USA [37, 38]. Two of the most frequent MSSA clones observed in our study (*spa* t002 and t008) were equivalent to MRSA strains and also to other commonly circulating ones in Europe [37]. These data suggest that the spread of *S*. *aureus* through hospitals and the community is related more to the characteristics of some clones than to methicillin resistance [39].

*Spa* types were equally distributed irrespective of the seriousness of the infection. Likewise, no specific *spa* type or clonal complex was implicated when cases of IE were considered. In the study by Nienaber *et al*., MSSA IE isolates were significantly more likely to be CC30 [40]. This result was not verified in our study, since none of the 8 CC30 were from IE cases. Tristan *et al*. also described a large diversity of *S*. *aureus* clonal complexes implicated in IE [41]. Feil *et al*. reported the molecular characteristics of 334 *S*. *aureus* and noted no link between MLST type and capability of triggering an infection [42]. Feil *et al*. explained their results by a weak link between clonality and virulence factors. In other words, isolates within the same lineage may be distinct due to their virulence factors and therefore their capability of infecting the patient.

Our study was not without limitations. First, it would have been interesting to characterize *S*. *aureus* strains found in 2012 in order to compare them to those of 2013 and distinguish any genetic differences particularly as the rate of *S*. *aureus* bacteremia was lower in 2012; however, they were not available. Furthermore, given that the type of molecular characterisation of strains concerns only a small part of the genome, our results give only a general overview of the genetic structure of our strain collection, albeit with a well-validated method [19]. Despite *spa* typing has been shown to be highly concordant with MLST [16, 17, 43, 44], *spa* CC t015 groups two distinct STs; *spa* type t015 has been related to ST45 (according to RIDOM database), while t571 is associated with ST398. One explanation given by Nübel *et al*. is that the sequence identity between *spa* types in different STs probably reflects the result of repeated convergent evolution of *spa* sequences on multiple occasions [45]. Due to the limitation on *spa* typing, the question of t571 attributed to CC398 has been solved by performing a specific PCR. Finally, the impact of the host’s genetic background has not yet been studied.

In conclusion, our results indicated that *S*. *aureus spa* t571 might be growing cause of bloodstream infections in our hospital. These results are concordant with other findings showing that *S*. *aureus* CC398, especially *spa* type t571 can cause invasive infections in humans. These strains appear to be highly receptive to horizontal gene transfer and acquisition of genetic elements leading to virulence and antibiotic resistance could occur. Therefore, careful monitoring of the evolution and epidemiology of *spa* t571 bacteremia is necessary. Further to this study, an infection control program of bacteremia was set up in Grenoble-Alpes University Hospital. Catheter related bacteremia are specifically targeted during weekly meeting gathering infectious disease specialists, bacteriologists and infection control specialists. The supervision is organized from the results of blood culture, in a prospective and continuous way over all year. The interpretation of these data constitutes the first stage of the follow-up of this infection, allowing the discussion of prevention measures.
